# Supplementary material for: Immunological Roles of TmToll-2 in Response to Escherichia coli Systemic Infection in Tenebrio molitor
Source: Int J Mol Sci. 2022 Nov 21;23(22):14490. doi: 10.3390/ijms232214490 (PMC9699188; doi:10.3390/ijms232214490)
Supplement: Supplementary file 1 [file ijms-23-14490-s001.zip › ijms-1981714-supplementary.pdf]

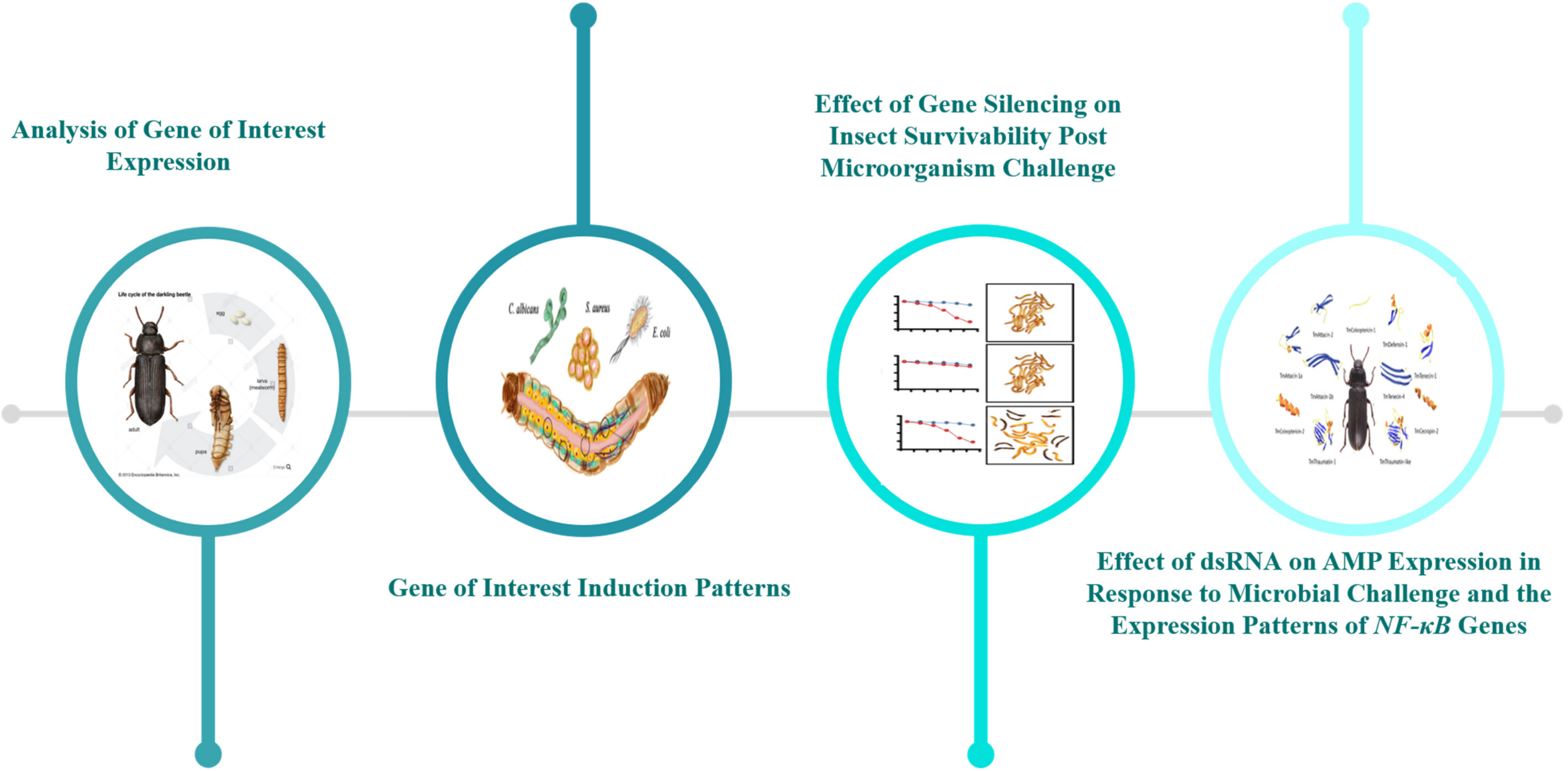

Figure S1. Schematic illustration of experimental procedure.

Following investigating the spatial and temporal expression of the *TmToll-2*, induction of mRNA expression of the gene has been analyzed. Further, innate immune response in *Tenebrio molitor* larvae has been studied after *TmToll-2* gene silencing by checking the AMP and NF-κB genes expression.
